# Statin therapy and mortality among new long-term care residents in Ontario, Canada: the contribution of clinical assessment data to a population-based cohort study

**DOI:** 10.23889/ijpds.v1i1.337

**Published:** 2017-04-19

**Authors:** Michael Campitelli, Susan Bronskill, Vasily Giannakeas, Andrew Morris, Colleen Maxwell, Chaim Bell

**Affiliations:** 1 Institute for Clinical Evaluative Sciences (ICES); 2 Mount Sinai Hospital; 3 University of Waterloo

## Objective

There is limited evidence from randomized trials and observational studies to guide clinical practice regarding the use of statins in long-term care (LTC); the effectiveness of statins among those with limited life expectancy is not clear and there is concern that the risk of drug-related adverse events might outweigh any benefit. We examined the impact of initiating statin therapy on mortality for patients newly admitted to LTC.

## Approach

Population-based health administrative data from Ontario, Canada were used to conduct a retrospective cohort study of newly admitted LTC residents, aged 66+ years and no statin use in the previous year, between January 1 2011 and December 31 2014. This cohort was linked to Resident Assessment Instrument (interRAI) data to capture clinical and functional characteristics (including frailty, activities of daily living, and cognitive function). The primary exposure was statin use within 30 days following LTC entry; residents who died or did not receive an interRAI assessment within 30 days were excluded. A propensity score for receiving statins was computed using resident demographic, clinical and functional characteristics. We matched exposed to unexposed patients on the basis of age (\pm1 year), sex, prior myocardial infarction(MI)/stroke hospitalization, frailty and propensity score (\pm0.2 standard deviations). Patients were followed in an intention-to-treat manner from the end of the exposure window until the earliest of death or March 31 2015. Cox regression was used to compare mortality between the study groups.

## Results

We identified 39,560 newly admitted LTC residents aged 66+ years with no statin use in the previous year, of which 1,953 (4.9%) were prescribed a statin within 30 days of LTC entry. Propensity score matching resulted in 1,710 pairs of exposed and unexposed patients. In the matched cohort, those receiving statins had a lower rate of mortality compared with those not receiving statins (Hazard Ratio 0.77; 95% Confidence Interval [CI] 0.70-0.85). In pre-specified subgroup analyses, the association between statin use and reduced mortality persisted among those with and without a prior MI/stroke hospitalization and among those categorized as frail and not frail.

## Conclusion

Our data suggest initiating statins may be beneficial in reducing mortality risk among LTC residents, despite the complexity and advanced age of the patients. By linking rich resident-level health and functional assessment data with health administrative data we were able to characterize the association between demographic and clinical characteristics (including frailty) and exposure to statins more fully than with administrative data alone.

